# Factors Associated with Zero-Dose Childhood Vaccination Status in a Remote Fishing Community in Cameroon: A Cross-Sectional Analytical Study

**DOI:** 10.3390/vaccines10122052

**Published:** 2022-11-30

**Authors:** Sangwe Clovis Nchinjoh, Yauba Saidu, Valirie Ndip Agbor, Clarence Mvalo Mbanga, Nkwain Jude Muteh, Andreas Ateke Njoh, Shalom Tchofke Ndoula, Bernard Nsah, Nnang Nadege Edwige, Sveta Roberman, Chen Stein Zamir

**Affiliations:** 1 Clinton Health Access Initiative Inc., Yaounde P.O. Box 2664, Cameroon; 2 Faculty of Medicine, The Hebrew University-Hadassah Braun School of Public Health and Community Medicine, Jerusalem 91120, Israel; 3 Institute for Global Health, University of Siena, 53100 Siena, Italy; 4 Nuffield Department of Population Health, University of Oxford, Oxford OX1 2JD, UK; 5 Gavi, The Vaccine Alliance, 1202 Geneva, Switzerland; 6 Expanded Program on Immunization, Cameroon Ministry of Public Health, Yaoundé P.O. Box 2084, Cameroon; 7 School of Global Health and Bioethics, Euclid University, Bangui BP 157, Central African Republic; 8 The Gordon Academic College of Education, Haifa 3570503, Israel

**Keywords:** zero-dose, childhood vaccination, Cameroon

## Abstract

**Background**: Cameroon’s suboptimal access to childhood vaccinations poses a significant challenge to achieving the Immunization Agenda 2030 goal—ranking among the top 15 countries with a high proportion of zero-dose (unvaccinated) children worldwide. There are clusters of zero-dose children in pockets of communities that traditionally miss essential healthcare services, including vaccination. The Manoka Health District (MHD) is home to such settlements with consistently low vaccination coverages (DPT-HepB-Hib-1: 19.8% in 2021) and frequent outbreaks of vaccine-preventable diseases (VPD). Therefore, the absence of literature on zero-dose children in this context was a clarion call to characterize zero-dose children in fragile settings to inform policy and intervention design. **Methodology**: This cross-sectional analytical study involved 278 children, 0–24 months of age, selected from a 2020 door-to-door survey conducted in the two most populous health areas in an archipelago rural district, MHD (Cap-Cameroon and Toube). We used R Statistical Software (v4.1.2; R Core Team 2021) to run a multivariable logistic regression to determine zero-dose associated factors. **Results**: The survey revealed a zero-dose proportion of 91.7% (255) in MHD. Children who were delivered in health facilities were less likely to be zero-dose than those born at home (AOR: 0.07, 95% CI: 0.02–0.30, *p* = 0.0003). Compared to children born of Christian mothers, children born to minority non-Christian mothers had higher odds of being zero-dose (AOR: 6.55, 95% CI: 1.04–41.25, *p* = 0.0453). Children born to fathers who are immigrants were more likely to be zero-dose children than Cameroonians (AOR: 2.60, 95% CI = 0.65–10.35, *p* = 0.0016). Younger children were likely to be unvaccinated compared to older peers (AOR: 0.90, 95% CI: 0.82–1.00, *p* = 0.0401). **Conclusions**: In the spirit of “leaving no child behind,” the study highlights the need to develop context-specific approaches that consider minority religious groups, immigrants, and younger children, including newborns, often missed during vaccination campaigns and outreaches

## 1. Background

In the past half century, morbidity and mortality due to vaccine-preventable diseases (VPD) have reduced tremendously in children [1]. This is primarily because of the substantial progress in vaccination coverage worldwide since the creation of the Expanded Program on Immunization (EPI) in 1974 [2]. In addition to the eradication of smallpox, the recent certification of the African Region as wild poliovirus-free, making it the fifth of six World Health Organization (WHO) regions, is another excellent example of the impact of effective vaccination [3]. Despite successes in global immunization, an estimated 21.8 million infants worldwide are still not being reached by routine immunization services [4]. Among the 19.7 million children worldwide who did not complete the three-dose of Diphtheria, Tetanus, and Pertussis-containing vaccine (DTP) series in 2019, 13.8 million (70%) were zero-dose children [2]. This number has witnessed a steep rise following the abrupt and rapid progression of the COVID-19 pandemic, which has significantly disrupted essential health service delivery in many countries, reversing past efforts to improve health indicators, including childhood immunization [5,6,7]. In 2021, about 25 million infants did not receive basic vaccines (the highest number since 2009), and the number of completely unvaccinated children (the so-called zero-dose children) increased by 5 million since the onset of the COVID-19 pandemic in 2019 [8].

Although many low- and medium-income countries (LMIC) have seen a steady increase in national-level vaccination coverage, many did not reach the 90% target for 2020 established by the World Health Organization (WHO) [9]. In fact, an estimated 20% of children in the African region are under-vaccinated despite the mammoth benefits of vaccination [10]. As a result, about three million children die annually of infectious diseases in this region, most of which are preventable by vaccination [10,11]. This is mainly due to suboptimal vaccination coverage in hard-to-reach subpopulations [12]. Therefore, achieving universal coverage with all recommended vaccines will require tailored, context-specific strategies to reach communities with substantial proportions of zero-dose and under-vaccinated children, particularly those in remote rural, poor semi-urban, conflict, and fragile settings [13].

In Cameroon, the EPI is responsible for childhood immunization, which is free for children under two years of age as shown in Table 1 below [14]. A household Demographic Health Survey (DHS) conducted in 2018 reported an immunization coverage (both from declaration and proofs of vaccination) of 86.7%, 71.5%, and 65.3% for Bacilli Calmette-Guérin (BCG), DTP-3, and measles-containing vaccines (MCV), respectively, with a zero-dose proportion of 9.7% [14]. The significantly low immunization coverage most likely explains the increase in reported cases of VPDs in Cameroon [15,16]. To reach global coverage goals with vaccines recommended across the life course, hard-to-reach and hard-to-vaccinate populations must be at the center of vaccination interventions [17]. The Manoka Health District (MHD) in the Littoral Region of Cameroon is one of such hard-to-reach districts with low vaccination coverage and several poorly documented outbreaks of VPDs. In 2021, the estimated DTP-1 vaccination coverage in MHD from the District Health Information Software 2 (DHIS2) was 19.8%, which is far below the 90% mark adopted by the Cameroon Ministry of Public Health (MoPH) during the World Health Assembly in 2012 [18].

Several studies in Cameroon have attempted to describe factors associated with incomplete vaccination and low vaccination coverage [14,17,19,20,21]. These factors include non-utilization of antenatal care services, younger mothers, being the ≥3rd born child in the family, lack of access to vaccination information, and longer distances from vaccinating facilities [17,22]. However, these studies are primarily hospital-based, conducted in urban settings, and did not characterize unvaccinated children living in pockets of communities that traditionally miss primary healthcare services, including immunization—the so-called missed communities. Therefore, this study aimed to close the knowledge gap on factors associated with zero-dose vaccination status among children 0–2 years of age in a missed community in Cameroon. These findings can be leveraged to inform policy and to design tailored programs to reduce the zero-dose proportion in the MHD and similar settings.

## 2. Methodology

### 2.1. Study Design and Setting

The study design was a cross-sectional analytical study. It was conducted in MHD, in the Littoral Region of Cameroon, over 20 km from Douala city. It is an enclaved archipelago district with about 19,943 persons distributed unequally across 47 islets. Most of the inhabitants are peasant fishermen who live in temporal houses with large family sizes of 5–12 persons. The men spend most of their time at sea fishing, while the women spend time at home with the children—their principal activities being fish ‘smoking’ and household chores. The island’s population comprises native Cameroonians and people from other countries (like Nigeria and Mali) who migrated for fishing. Immigrants make up over 70% of the total population. Most of these immigrants lack a residence permit that grants them legal status to live in Cameroon, limiting the freedom to travel to other towns/cities for essential commodities and services, including health services. Therefore, they depend on local boat couriers to purchase goods from out-of-town, and roadside drug vendors, dispensaries, and traditional healers for their health care. A single health facility serves the entire district. Pregnant women mostly deliver at home in the hands of traditional birth attendants and relatives, resulting in a considerable proportion of unregistered live births. Consequently, children in this district are generally missed by routine vaccination and other primary health care services. 

### 2.2. Data Collection and Sampling

This study was based on secondary data collected in 2020 by the Clinton Health Access Initiative (CHAI) in partnership with Gavi, the vaccine alliance, and the Cameroon EPI. During this period, they conducted a door-to-door survey in MHD to identify zero-dose and under-immunized children. The field team employed convenience sampling to select health areas (administrative level 4) in MHD for the survey based on population size. Trained community health workers (CHWs) used convenience sampling to administer structured survey tools to caregivers based on their availability in the two most populous health areas (Cap-Cameroon and Toube)—the combined population constitutes over a third of the entire population of the MHD. Data captured were primarily vaccination status and relevant socio-demographic factors, such as the parent’s level of education, religion, educational level, sex, age, and place of delivery. 

The vaccination status of children was based on caregivers’ recall because it was realized that most children were only vaccinated during Supplementary Immunization Activities (SIA) and vaccination campaigns—in the past five years, vaccination cards were only issued during routine immunization. The team, therefore, decided to rely on caregiver recall to avoid losing valuable data due to the exclusion of children without vaccination cards and birth certificates. Moreover, data collectors corroborated caregivers’ information on children’s vaccination status with a checklist containing the timing of SIA and vaccination campaigns conducted in MHD in the past five years to minimize bias. All children under two years of age surveyed in the zero-dose identification project in MHD were considered for the study. However, children whose caregivers were unable to recall whether they were vaccinated were excluded. 

### 2.3. Key Operational Definitions

Where vaccination cards or birth certificates were unavailable, the study relied on caregivers’ recall to attribute the vaccination status of children. The outcome of interest was zero-dose vaccination status. Zero-dose children were those who had never received any recommended vaccine antigen for their age based on the Cameroon EPI calendar. Completely vaccinated children included those who had received all vaccines recommended for their age. Incompletely vaccinated children were those who had received at least one dose of any of the recommended vaccines but had not completed vaccines that were appropriate for their age. Finally, at least single-dose (ASD) vaccinated children were those who had received at least one dose of any vaccine. Therefore, ASD encompassed both incomplete and completely vaccinated children.

### 2.4. Data Management and Analysis

The zero-dose project dataset on children born between 31/11/2018 and 30/08/2020 was exported as a Microsoft Excel 2013 worksheet into R Statistical Software (v4.1.2; R Core Team 2021) for statistical analysis. Categorical variables (sex, birth site, vaccination status, availability of birth certificate, health area (administrative level 4), child’s birth order, marital status, parents’ educational level, occupation, religion, and nationality) were summarized in percentages. Collinearity was evaluated for predictor variables before including them in the final regression model. Missing data points were included in the analysis. Univariate analysis was used to determine associations between individual explanatory variables and the zero-dose vaccination status of children. The factors independently associated with zero-dose vaccination status and explanatory variables with *p* < 0.2 in univariate analysis were included in the multivariate logistic regression with zero-dose status as an outcome. The adjusted odd ratios (AOR) with corresponding 95% Confidence Intervals (CI) were then calculated. The decision to use explanatory variables with *p* < 0.2 in the univariate analysis as factors in the multivariate model was to maximize the chance of capturing variables that might have an effect on the association or explain some of the variances in the outcome, even though they were not significantly associated with it. To verify the robustness of our results, they were compared to those obtained from a multivariable model that includes all potential explanatory variables as factors. 

### 2.5. Ethical Considerations

CHAI had obtained ethical clearance from the Cameroon National Ethics Committee before data collection. Authorization from appropriate CHAI Cameroon administrative authorities was obtained to gain access to the zero-dose project dataset. Furthermore, the data was used solely for this study and not shared with any third party. 

## 3. Results

During the zero-dose identification, head counting of children under two was conducted in all households (100%) in the two most populous islets (Cap-Cameroon and Toube)—284 children under two years of age were identified. However, six children were excluded from the analysis because their parents were unavailable during the survey, and the available relatives could not provide information about their vaccination status. 

The mean age of children included in the study was 11.6 months (SD = ±6.7), with male children overrepresented (53.2%). A considerable proportion of the children (92.8%) were born at home with the aid of traditional birth attendants. As such, most of them had no proof of dates of birth (birth certificates), 93.5%, making information on child age heavily reliant on the caregiver’s recall. Children had immigrant mothers for the most part (87.1%) and were mainly from the Cap-Cameroon health area (57.6%).

Of the 278 children retained for the final analysis, 8.3% were ASD children (1.8% completely vaccinated and 6.5% incompletely vaccinated), while 91.7% were zero-dose cases. ASD cases were mainly vaccinated during a national vaccination campaign (78.3%), with BCG being the most utilized antigen (73.9%). The details of the socio-demographic characteristics of all the children surveyed, the distribution of zero-dose children by socio-demographic factors, and the chi-square test results are outlined in Table 2 below. Table 3 highlights the vaccination profile of the surveyed children. This univariate analysis revealed that the child’s age, birth site, owning a birth certificate, and nationality of both parents are significantly associated with zero-dose vaccination status. There was no significant difference in the distribution of zero-dose children among the variables of the other socio-demographic factors. However, the birth order, mother’s age, mother’s religion, and mother’s educational level were included in the multivariate analysis based on *p* < 0.2 to increase the chance of capturing variables that might be associated with the zero-dose vaccination status.

### Factors Associated with Zero-Dose Vaccination Status

Table 4 highlights multivariable logistic regression results to determine factors independently associated with being a zero-dose child. Factors associated with zero-dose vaccination status are younger children, children born at home, children born of immigrant fathers, and of non-Christian mothers. As seen from the Table, the odds of being a zero-dose child decrease with the child’s age and birth in a health facility, but increase among children born to immigrant fathers or non-Christian mothers. Children delivered in health facilities are less likely to be zero-dose than those born at home (AOR: 0.07, 95% CI: 0.02–0.30, *p* = 0.0003). Similarly, compared to a Christian mother, children born to minority non-Christian mothers have higher odds of being zero-dose (AOR: 6.55, 95% CI: 1.04–41.25, *p* = 0.0453). Children born to fathers who are non-nationals are likelier to be zero-dose children than Cameroonians (AOR: 2.60, 95% CI = 0.65–10.35, *p* = 0.0016). Also, younger children are likely to be unvaccinated compared to older peers (AOR: 0.90, 95% CI: 0.82–1.00, *p* = 0.0401).

## 4. Discussion

This research reveals a zero-dose proportion of 91.7% (255/278), almost ten times the Cameroon national zero-dose proportion of 9.7% reported in 2020 [23]. This low vaccination coverage contributing to the low national EPI coverage could be explained partly by factors peculiar to its hard-to-reach characteristics. These factors include the absence of health facilities in the study health areas, distance from the lone health facility, multiple poorly accessible communities (islets), and frequent diurnal flooding, making access an uphill task. Our findings are consistent with a publication by Ozawa et al. in 2019 on the characteristics of hard-to-reach communities—based on an extensive literature review from 2000 to 2018 [24]. In our study, the primary service delivery approach employed in vaccinating children was mass vaccination campaigns—78.3% of vaccinated children were vaccinated through this approach. This highlights the importance of Supplementary Immunization Activities (SIA) and vaccination campaigns in improving vaccination coverage in hard-to-vaccinate communities, similar to the role of SIA in preventing measles outbreaks during the COVID-19 pandemic in Kenya [25]. 

Based on the multivariable logistic regression analysis, the log odds of being a zero-dose child decreased with the child’s age and being born in a health facility. However, children born to immigrant fathers and non-Christian mothers had higher odds of being zero-dose children than those born to Cameroonian fathers and Christian mothers, respectively. Younger children are likely to be unvaccinated compared to their older peers (AOR: 0.90, 95% CI: 0.82–1.00, *p* = 0.0401). This can be explained by the fact that this population depends solely on outreach and mobile strategies for vaccination and most often have to wait for a national vaccination campaign or an interventional vaccination program during an epidemic to receive routine vaccines. By reviewing demographic and health surveys in sub-Saharan Africa, Mutua et al. showed that on-time vaccination was relatively low in sub-Saharan Africa and varied depending on different factors, including place of residence [1]. This implies younger children are likely to miss their vaccines and only get them at an older age. This is also consistent with a study by Stein-Zamir et al. in Israel, which showed that age-specific vaccine delays would lead to fewer vaccination cases at younger ages compared to older children [26]. A study in 2018 showed the impact of a mobile vaccination strategy in hard-to-reach communities, with children of older ages having higher vaccination coverages than those of younger age groups, similar to the findings in this study [27]. It is, therefore, of significant value to design tailored approaches that permit routine vaccination of children from birth to ensure all children benefit from vaccine protection throughout childhood.

Children born in health facilities were less likely to be unvaccinated than those delivered at home (AOR: 0.07, 95% CI: 0.02–0.30, *p* = 0.0003). Since women deliver at home, they miss the vaccines given to the child at birth, including vaccination-related counseling and scheduling, which can explain this finding. Also, 93.5% of children in this study did not have birth certificates, which presents a challenge in determining a child’s age, posing a problem in terms of logistics, routine vaccination micro-planning, and the vaccination activity itself, as it relies on the ages of the children. This is consistent with other studies, though they did not focus on zero-dose cases, but were more interested in incomplete and complete vaccination cases [24,28,29,30]. In missed communities, a context-specific approach, such as setting up micro-health facilities or collaborating with traditional birth attendants to identify, track and vaccinate children from birth, will significantly improve immunization coverage and the fight against VPDs.

Children born to immigrant fathers were likely to be zero-dose children compared to children whose fathers were native Cameroonians (AOR: 2.60, 95% CI = 0.65–10.35, *p* = 0.0016). Most immigrants do not have a residence permit and as such, they cannot easily access essential health services outside their current residence. As a result, they tend to depend on traditional healers, birth attendants, roadside drug vendors, and unregistered private dispensaries for their healthcare needs. As such, even if parents are willing to vaccinate their children, they would have no choice but to wait for an outreach vaccination program since they cannot travel to get vaccines outside of this setting. A systemic review of studies in sub-Saharan Africa and the European region revealed migration as a factor associated with low vaccination coverage [31,32]. Also, comparatively lower vaccination coverage was found among immigrants in India compared to the locals because of the high prevalence of home births, lack of awareness of the location of health facilities, mobility, and fear of vaccine side effects [33]. 

In the same line, this study reveals that children born to minority non-Christian mothers are likelier to be zero-dose children than those born by Christian mothers (AOR: 6.55, 95% CI: 1.04–41.25, *p* = 0.0453). The non-Christian communities in MHD represent a minority population, with only 5.4% of mothers belonging to this population as opposed to their Christian counterparts, 93.8%. To leave no child unvaccinated, the finding in this study further emphasizes the need to identify minority communities; employ human-centered design and tools, such as the WHO framework of behavioral and social drivers (BeSD), to have in-depth knowledge on supply and demand barriers specific to minority populations; and develop context-specific strategies.

Unlike most studies, birth order was not significantly associated with zero-dose vaccination status, AOR, 1.33, 95% CI: 0.97–1.81, *p* = 0.0753 [28,34,35]. For instance, a nested case-control study conducted on a cohort of 110,902 Israeli children under the age of 5 revealed that birth order progression is inversely associated with vaccine utilization [36]. The critical explanation is that previous parental vaccination service delivery experiences with their firstborns tend to shape parents’ new attitudes towards vaccination [36]. Birth order was probably insignificant in this study because vaccination coverage was too low in these health areas to significantly impact subsequent parental attitudes toward vaccination.

## 5. Conclusions

This study establishes an association between being a zero-dose child and home-based births, being the younger child, being born to immigrant fathers, and minority non-Christian mothers. Therefore, the study highlights the need to develop context-specific approaches to vaccinating children in hard-to-reach communities to close health equity gaps. This can be achieved by paying more attention to minority groups, immigrants, and younger children, including newborns, who are often missed during vaccination campaigns. The study findings also reemphasize the value of SIA in such a missed community.

### 5.1. Limitations

A major flaw in this study is the possibility of non-differential misclassification of the vaccination status of children since more than 90% of children did not have birth certificates, and their ages were estimated based on their parents’ recall. Although this may have affected the proportion of incomplete and complete vaccination cases, it did not affect the multivariable logistic regression findings because the outcome variable was solely based on whether the child had ever received any vaccine antigen on the Cameroon EPI calendar (zero-dose vaccination status). Also, we minimized bias stemming from the caregiver’s recall by corroborating the children’s vaccination status with a checklist of timing of SIA and national vaccination campaigns in the past five years.

The certainty of the evidence is limited by the small sample size of specific populations, such as non-Christian mothers and fathers, and the number of health facility-based births. Apart from the limited statistical power of this study, the cross-sectional study design conducted using secondary data posed a challenge of generalizability. However, the findings are aligned with many similar studies in other countries.

Convenience sampling was employed which, may have compromised the generalizability of this study. However, the sampling approach took into consideration the most populous islets in MHD with high zero-dose proportion. The population dynamics and social activities of these communities make availability a major issue—this is the reason why convenience sampling was a great fit so as not to lose valuable data. 

### 5.2. Recommendation

A qualitative study to establish in-depth reasons for zero-dose and under-vaccinated children will further close the knowledge gap on missed communities in Cameroon.

## Figures and Tables

**Table 1:** Cameroon EPI Calendar.

Contacts	Age	Antigenes *
1st contact	At birth	BCG, OPV 0
2nd contact	Six weeks	Penta 1 (DPT-1 + Hep B1 + HIB1), OPV-1, Pneumo 13-1, Rota-1
3rd contact	Ten weeks	Penta 2 (DPT-2 + Hep B2 + HIB2), OPV-2, Pneumo 13-2, Rota-2
4th contact	14 weeks	Penta 3 (DPT-3 + Hep B3 + HIB3), OPV-3, Pneumo 13-3, IPV
5th contact	Nine months	MR1, yellow fever vaccine
6th contact	15 months	MR2

* BCG = Bacillus Calmette-Guerin, Penta = Pentavalent vaccine, DPT = Diphtheria, Pertussis, and Tetanus vaccine, Hep B1 = Hepatitis B vaccine, HIB = *Haemophilus influenzae* vaccine, OPV = Oral Polio vaccine, Pneumo 13 = Pneumococal 13 valent conjugate vaccine, IPV = Injectable polio vaccine.

**Table 2:** Distribution of zero-dose vaccination status by socio-demographic factors.

Characteristic	N = 278	Zero-Dose (%)	*p*-Value
Sex			0.7453
Male	148 (53.2%)	137 (92.6%)	
Female	129 (46.4%)	118 (91.5%)	
Health Area			0.5781
Cap-Cameroon	160 (57.6%)	145 (90.1%)	
Toube	118 (42.4%)	110 (93.2%)	
Birth Site			0.0000
Home	258 (92.8%)	244 (94.6%)	
Health facility	20 (7.2%)	11 (55%)	
Birth Certificate			0.0077
Yes	18 (6.5%)	13 (72.2%)	
No	260 (93.5%)	242 (93.1%)	
Father’s nationality			0.0000
Cameroonian	46 (16.5%)	33 (71.7%)	
Immigrants	211 (75.9%)	202 (95.7%)	
Father’s Profession			0.8702
Fishing	220 (79.1%)	202 (91.8%)	
Others	38 (13.7%)	34 (89.4%)	
Father’s Religion			0.7748
Christian	241 (86.7%)	221 (91.7%)	
Others	14 (5.0%)	12 (85.7%)	
Father’s Educational level			0.8450
Not Educated	182 (65.5%)	166 (91.2%)	
Primary Education	46 (16.5%)	43 (93.5%)	
Secondary Education	30 (10.8%)	27 (90%)	
Mother’s nationality			0.0000
Cameroonian	35 (12.6%)	25 (71.4%)	
Immigrant	242 (87.1%)	229 (94.6%)	
Mother’s Profession			0.5431
Housewife	167 (60.1%)	155 (92.8%)	
Others	110 (39.6%)	99 (90%)	
Mother’s Religion			0.0623
Christian	256 (92.1%)	237 (92.6%)	
Non-Christians	15 (5.4%)	13 (86.7%)	
Mother’s Educational level			0.1589
None	104 (37.4%)	98 (94.2%)	
Primary Education	135 (48.6%)	124 (91.9%)	
Secondary Education	38 (13.7%)	32 (84.2%)	
Marital Status of Mother			1
Married	242 (87.1%)	222 (91.7%)	
Others	33 (11.9%)	30 (90.9%)	

**Table 3:** Vaccination status of the children surveyed.

Characteristic	Frequency (%)
Vaccination Status (N = 278)	
Complete	5 (1.8)
Incomplete	18 (6.5)
Zero-dose	255 (91.7)
Vaccination site for ASD (N = 23)	
Manoka District Hospital	17 (73.9)
Other Districts	5 (21.7)
Vaccination strategy for ASD (N = 23)	
Vaccination campaigns	18 (78.3)
Routine vaccination in a health facility	4 (17.4)
Antigen received by ASD (N = 23)	
BCG	17 (73.9)
OPV 0	13 (56.5)
OPV 1	5 (21.7)
OPV 2	4 (17.4)
OPV 3	3 (13)
IPV	3 (13)
Penta 1, PCV 1	4 (17.4)
Penta 2, PCV 2	4 (17)
Penta 3, PCV 3	3 (13)
Rota 1	4 (17.4)
Rota 2	2 (8.7)
Measles/yellow fever	0 (0.0)
Reason for non-vaccination among zero-dose children (N = 255)	
No health facility	234 (91.7)
No transportation means	10 (3.9)
No reason	11 (4.3)

**Table 4:** Factors associated with zero-does status.

		Univariate Logistic Analysis			Multivariate Logistic Analysis	
	N	Zero-Dose (%)	COR (95%CI)	*p* Value	AOR (95% CI)	*p* Value
Age of child (months)	-	-	0.91 (0.84–0.97)	0.0074	0.90 (0.82–1.00)	0.0401
Child’s birth order	-	-	1.27 (1.01–1.60)	0.0426	1.33 (0.97–1.81)	0.0753
Birth site						
Home	258	94.6	1		1	
Health facility	20	55	0.07 (0.02–0.20)	0.0000	0.07 (0.02–0.30)	0.0003
Birth Certificate						
No	18	72.2	1		1	
Yes	260	93.1	0.19 (0.06–0.60)	0.0046	0.76 (0.15–3.85)	0.7354
Mother’s educational level						
Not Educated	104	94.2	1		1	
Primary Education	135	91.9	0.69 (0.25–1.93)	0.4802	0.76 (0.19–2.93)	0.6903
Secondary Education	38	84.2	0.33 (0.01–1.08)	0.0675	0.53 (0.09–2.96)	0.4692
Mother’s Religion						
Christian	256	96.2	1		1	
Non-Christians	15	86.7	0.26 (0.08–0.88)	0.0299	6.55 (1.04–41.25)	0.0453
Mother’s Nationality						
Cameroonian	35	71.4	1		1	
Immigrants	242	94.6	7.05 (2.80–17.72)	0	2.60 (0.65–10.35)	0.1760
Father’s Nationality						
Cameroonian	46	71.7	1		1	
Immigrants	211	95.7	8.84 (3.50–22.32)	0	8.92 (2.29–34.65)	0.0016
Mother’s Age	--	--	1.01 (0.95–1.07)	0.7362	1.00 (0.92–1.09)	0.9794

## Data Availability

Data used for this research are available from the corresponding author upon reasonable request.
